# Management of Acute Lung Injury: Palmitoylethanolamide as a New Approach

**DOI:** 10.3390/ijms22115533

**Published:** 2021-05-24

**Authors:** Alessio Filippo Peritore, Ramona D’Amico, Rosalba Siracusa, Marika Cordaro, Roberta Fusco, Enrico Gugliandolo, Tiziana Genovese, Rosalia Crupi, Rosanna Di Paola, Salvatore Cuzzocrea, Daniela Impellizzeri

**Affiliations:** 1 Department of Chemical, Biological, Pharmaceutical and Environmental Science, University of Messina, 98122 Messina, Italy; aperitore@unime.it (A.F.P.); rdamico@unime.it (R.D.); rsiracusa@unime.it (R.S.); rfusco@unime.it (R.F.); tgenovese@unime.it (T.G.); dimpellizzeri@unime.it (D.I.); 2 Department of Biomedical and Dental Sciences and Morphofunctional Imaging, University of Messina, 98122 Messina, Italy; cordarom@unime.it; 3 Department of Veterinary Science, University of Messina, 98122 Messina, Italy; egugliandolo@unime.it (E.G.); rcrupi@unime.it (R.C.); 4 Department of Pharmacological and Physiological Science, Saint Louis University School of Medicine, Saint Louis, MO 63104, USA

**Keywords:** palmitoylethanolamide, ultramicronized, inflammation, acute lung injury, cytokines

## Abstract

Acute lung injury (ALI) and acute respiratory distress syndrome (ARDS) are common and devastating clinical disorders with high mortality and no specific therapy. Lipopolysaccharide (LPS) is usually used intratracheally to induce ALI in mice. The aim of this study was to examine the effects of an ultramicronized preparation of palmitoylethanolamide (um-PEA) in mice subjected to LPS-induced ALI. Histopathological analysis reveals that um-PEA reduced alteration in lung after LPS intratracheal administration. Besides, um-PEA decreased wet/dry weight ratio and myeloperoxidase, a marker of neutrophils infiltration, macrophages and total immune cells number and mast cells degranulation in lung. Moreover, um-PEA could also decrease cytokines release of interleukin (IL)-6, interleukin (IL)-1β, tumor necrosis factor (TNF)-α and interleukin (IL)-18. Furthermore, um-PEA significantly inhibited the phosphorylation of nuclear factor of kappa light polypeptide gene enhancer in B-cells inhibitor, alpha (IκBα) and nuclear factor kappa-light-chain-enhancer of activated B cells (NF-κB) activation in ALI, and at the same time decreased extracellular signal-regulated kinase 1/2 (ERK1/2), c-Jun N-terminal kinase (JNK) and p38 mitogen-activated protein kinase (p38/MAPK) expression, that was increased after LPS administration. Our study suggested that um-PEA contrasted LPS-induced ALI, exerting its potential role as an adjuvant anti-inflammatory therapeutic for treating lung injury, maybe also by p38/NF-κB pathway.

## 1. Introduction

Acute lung injury (ALI) represents the most severe form of the viral infection sustained by coronavirus disease 2019 (COVID-19). To date, in an ongoing pandemic condition, although several compounds have been proposed as a treatment, none of them can be considered a definitive solution. Among patients with severe COVID-19, some have shown laboratory evidence of an inflammatory response, similar to the cytokine release syndrome, with elevated pro-inflammatory cytokines and persistent fevers; such patient profiles have been related with serious and fatal illnesses [1,2]. ALI and acute respiratory distress syndrome (ARDS) are the main causes of acute respiratory failure which are associated with considerable morbidity and mortality [3]. ALI is characterized by the disruption of endothelium and alveolar epithelial barrier, leading to increased microvascular permeability. Inflammatory response plays a central role in the development of ALI, which occurs through the signal transduction pathways to activate inflammatory cells. In the lung, the pathological transition leading to the overproduction of pro-inflammatory cytokines is characterized by diffuse alveolar damage accompanied by neutrophilic infiltration and protein-rich edema in the alveolar space [4]. The inflammation induced by these immune cells is modulated by the inflammatory mediators, including interleukin (IL)-1β, interleukin (IL)-6, tumor necrosis factor (TNF)-α and interleukin (IL)-18 [5]. In the pathogenesis of viral infections, among immune cells, mast cells (MCs) play an important role as inflammatory mediators. Various pathogens, such as viruses, bind MCs through the toll-like receptor (TLR), a bond that activates cells by promoting their degranulation and thus the release of pro-inflammatory chemicals and cytokines. Although, in the scientific literature, there are only a few reported works on the activation of MCs following virus infection [6]. Following activation, mast cells release the contents of their granules, such as preformed mediators, including histamine, and above all, several specific proteases like chymase, tryptase and carboxypeptidase [7,8]. Activation of mast cells in the lungs also contributes to progression of allergy and inflammatory diseases on the respiratory system. Previous studies have shown that tryptase exhibits a proinflammatory function by stimulating inflammatory and structural cells, including neutrophils, eosinophils, epithelial and endothelial cells in the lung [9,10]. Furthermore, the expression of MC chymase has been seen to be increased in human pulmonary fibrosis [11]. Lipopolysaccharide (LPS), a component of the cell wall of Gram-negative bacteria, has been implicated in the pathogenesis of ALI [12]. LPS is a specific ligand of TLR4, and ALI is associated with the activation of LPS-induced TLR4 signaling pathways. Palmitoylethanolamide (PEA) is an endogenous lipid [13,14] contained in several foods, that belongs to the ALIAmides (Autocoid Local Injury Antagonism) family [15,16]. PEA has important analgesic and anti-inflammatory properties on numerous molecular targets [17,18], which showed important inhibitory effect against mast cell degranulation [18,19]. The anti-inflammatory action of PEA has been abundantly demonstrated in several animal models of inflammation, such as tuberculin hypersensitivity, adjuvant-induced arthritis, carrageenan-induced paw edema and ischemia reperfusion injury [20,21,22]. Given the lipidic nature of PEA, different limits of solubility and bioavailability have been found; to overcome this problem, an ultramicronization technique has been applied to decrease the size of the molecule and increase the dissolution rate [23] while reducing variability of absorption. Recent studies have proposed um-PEA as a potential adjunct in therapy for COVID patients, thanks to its ability to modulate inflammation and neuroinflammation, both peripherally and centrally [24,25]. Precisely these pleiotropic properties could be taken into consideration for the introduction of um-PEA in the COVID-19 multidrug regimen, thus avoiding an increase in the dosage of immunosuppressants by planning a synergistic therapy between um-PEA and the latter [26,27]. The mechanisms underlying ALI are still unclear, so in the present study, we used a model of intratracheally LPS-induced ALI to evaluate the effects of um-PEA 30 mg/kg on regulation of inflammatory process in acute lung disease. 

## 2. Results

### 2.1. Um-PEA Ameliorated the Histological Alteration and Decreased the Lung Edema and Neutrophils Activity

**Figure 1:**
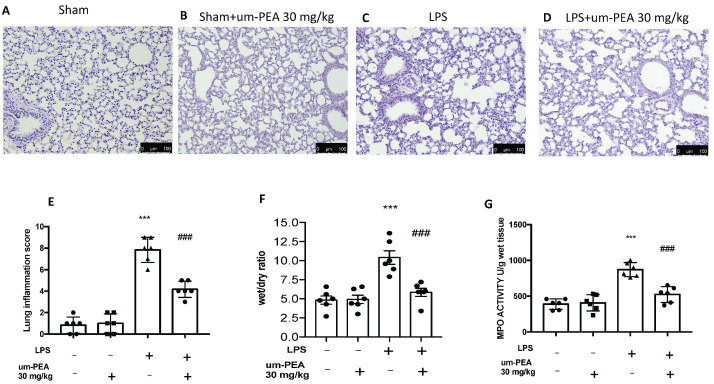
The effects of um-PEA on histological damage after LPS i.t. injection. Histological analysis was evaluated in sham (**A**), sham + um-PEA (**B**), LPS (**C**), LPS + um-PEA (**D**) groups. Lung inflammation scores were assessed from 0 to 5 points for all of the following parameters: alveolar congestion, hemorrhage, infiltration or aggregation of neutrophils in airspace or vessel wall, and thickness of alveolar wall/hyaline membrane formation (**E**), lung edema (W/D) (**F**) and MPO activity (**G**). Images are figurative of at least three independent experiments. Values = means ± SEM of six animals in each group; *** *p* < 0.001 vs. sham; ### *p* < 0.001 vs. LPS.

Haematoxylin and eosin (H & E) revealed the copious tissue damage and extracellular matrix deposition in lungs of LPS-treated mice compared to the sham groups (Figure 1A,C,E). In the same way, um-PEA oral administration significantly reduced injury in lungs (Figure 1D,E). In addition, no significant difference was observed in the sham+um-PEA (Figure 1B). ALI score was assessed as follows: alveolar congestion, hemorrhage, infiltration or aggregation of neutrophils in airspace or vessel wall, and thickness of alveolar wall/hyaline membrane formation. Each item score was scored on a five-point scale as follow: 0 = minimal damage, 1 = mild damage, 2 = moderate damage, 3 = severe damage, 4 = maximal damage (Figure 1E). ALI is associated to increase of lung edema, as shown by the ratio of wet/dry (W/D) weight of the tissue (Figure 1F). In order to assess the activity of neutrophils, we also performed the MPO assay. ALI increased the MPO activity, while um-PEA administration reduced MPO and ratio of W/D weight of the tissue (Figure 1G).

### 2.2. Um-PEA Modulates the Inflammatory Cells and Cytokines in Bronchoalveolar Lavage Fluid (BALF)

**Figure 2:**
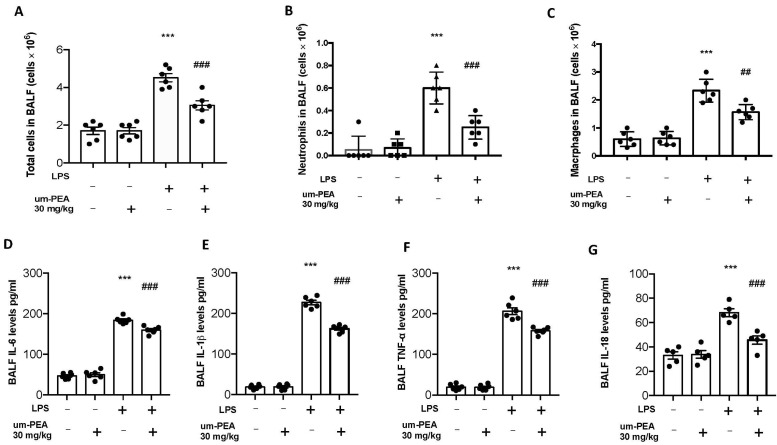
Effect of um-PEA on cell infiltration and proinflammatory cytokine expression in BALF: total cells (**A**); macrophages (**B**) and neutrophils (**C**); IL-6 (**D**); IL-1β (**E**); TNF-α (**F**) and IL-18 (**G**) levels in BALF. Values = mean ± SEM of six animals in each group; *** *p* < 0.001 vs. sham, ## *p* < 0.01 vs. LPS and ### *p* < 0.001 vs. LPS.

Twenty-four hours after LPS instillation, um-PEA administration reduced the number of inflammatory cells in the BALF; in particular, we evaluated total cells, macrophages, and neutrophils, observing a significant rise in cell numbers in BALF collected from LPS-treated animals compared to the sham groups. No significant difference was observed between the sham and sham + um-PEA groups in term of inflammatory cells in BALF (2A–C). At the same time, um-PEA oral administration was able to reduce the number of inflammatory cells in BALF (Figure 2A–C). To test whether um-PEA treatment regulated the inflammatory process through modulation of cytokines release, we evaluated the BALF levels of the pro-inflammatory cytokines IL-6, TNF-α, IL-1β and IL-18. A meaningful increase of IL-6, TNF-α, IL-1β and IL-18 secretion was observed in BALF of LPS-treated mice compared with sham group animals. In contrast, it was observed a significant inhibition of IL-6, TNF-α, IL-1β and IL-18 in mice treated with um-PEA (Figure 2D–G). Moreover, no significant difference was observed in terms of cytokines release in the sham+um-PEA group compared to sham (2D–G).

### 2.3. Um-PEA Reduced Mast Cell Degranulation

**Figure 3:**
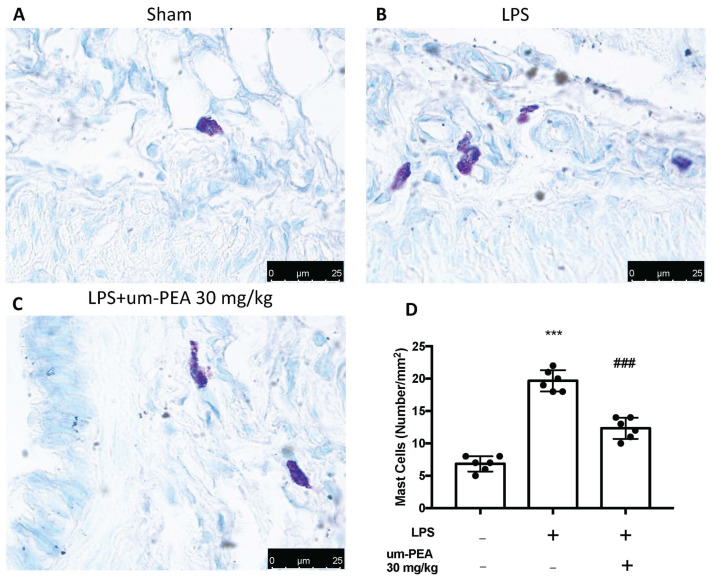
The presence of mast cells is indicated by toluidine blue staining: Sham (**A**), LPS (**B**), LPS + um-PEA (**C**); mast cell count (**D**) in lungs. Figures are representative of at least 3 independent experiments. Values are means SEM of 6 animals for each group; *** *p* < 0.001 vs. sham, ### *p* < 0.001 vs. LPS.

**Figure 4:**
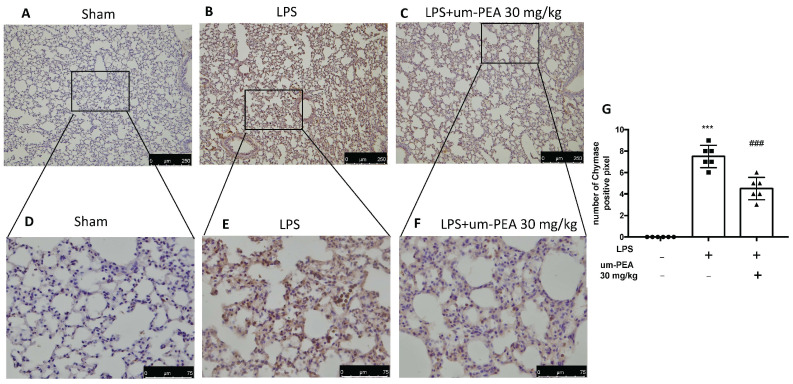
Effect of um-PEA on chymase: Sham (**A**), LPS (**B**), LPS+um-PEA (**C**) for image 10× magnification, and Sham (**D**), LPS (**E**), LPS+um-PEA (**F**) for 40× magnification in lungs. The results are expressed as % of positive pixels (**G**). Images are figurative of at least three independent experiments. Values = means ± SEM of six animals in each group; *** *p* < 0.001 vs. sham, ### *p* < 0.001 vs. LPS.

**Figure 5:**
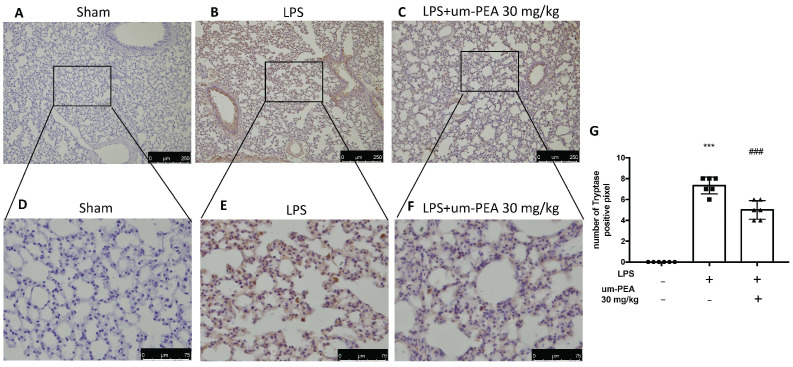
Effect of um-PEA on tryptase: Sham (**A**), LPS (**B**), LPS+um-PEA (**C**) for image 10× magnification, and Sham (**D**), LPS (**E**), LPS + um-PEA (**F**) for 40× magnification in lungs. The results are expressed as % of positive pixels (**G**). Images are figurative of at least three independent experiments. Values = means ± SEM of six animals in each group; *** *p* < 0.001 vs. sham, ### *p* < 0.001 vs. LPS.

Evaluation of mast cell activation was performed by toluidine blue staining of lung tissues. LPS-treated animals displayed an increased number of infiltrating mast cells compared to the sham animals (Figure 3A,B,D). um-PEA treatment reduced the recruitment in lungs (Figure 3C,D). LPS administration also increased chymase activity in lungs compared to sham animals, while um-PEA was able to reduce it (Figure 4). Moreover, LPS was able to increase tryptase in mice after 24 h compared to sham animals, while um-PEA prevented the tryptase increase (Figure 5).

### 2.4. Um-PEA Manages the pERK, pJNK, p38MAPK and NF-kB Pathway

**Figure 6:**
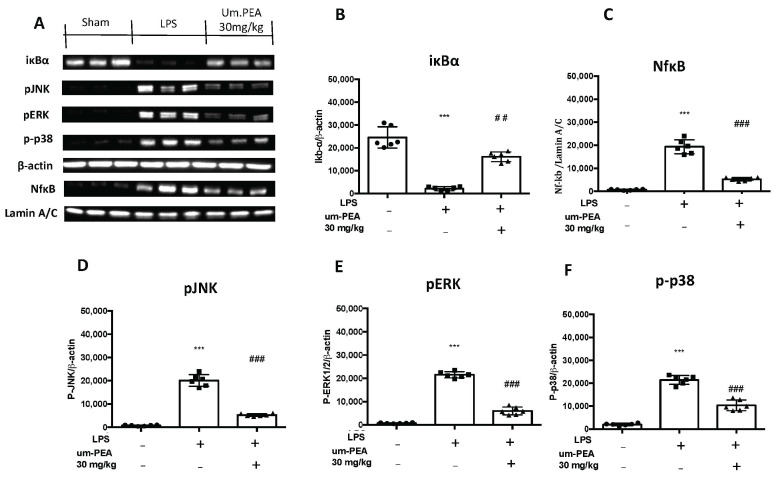
Western blots for IKB-α, NF-kB, pJNK, pERK1/2 and p38MAPK, (**A**). Representative Western blots for cytoplasmic IKB-α degradation (**A**,**B**), nuclear NF-κB translocation (**A**,**C**), cytoplasmic pJNK (**A**,**D**), cytoplasmic pERK1/2 (**A**,**E**) and cytoplasmic p-p38 (**A**,**F**). A demonstrative blot of lysates (six animals/group) with a densitometric analysis for all animals is shown. The results in (**B**–**F**) correspond to means ± SEM of six animals in each group; *** *p* < 0.01 vs. sham, ### *p* < 0.001 vs. LPS.

We investigated one of the key inflammatory pathways induced by LPS-induced ALI: the pERK, pJNK, p-p38 and Ikb-α/NF-kB systems. Ikb-α basal expression was observed in lungs collected from sham mice, while LPS instillation reduced it (Figure 6A,B). In addition, NF-kB expression in the nucleus was enhanced in LPS-treated mice compared to the sham (Figure 6A,C). Treatment with um-PEA significantly increased Ikb-α cytosol expression (Figure 6A,B) and decreased NF-kB expression in the nucleus (Figure 6A,C). In the cytoplasm, p-JNK expression levels (Figure 6A,D), monitored by Western blotting, such as p-ERK (Figure 6A,E) and p38MAPK (Figure 6A,F), were considerably increased in tissue collected from LPS-treated mice compared to the sham animals. Lung tissues collected from um-PEA-treated mice showed a significant reduction in the cytoplasm of p-JNK, as well as p-ERK and p38MAPK expression (Figure 6A,D,F).

## 3. Discussion

ALI is an acute inflammatory condition, which can further progress to an austere stage called ARDS, characterized by a high incidence rate of mortality [28]. Intrapulmonary and extrapulmonary factors are the causes into which acute lung injury are divided clinically [29]. Among the exogenous lung factors are included burns, pus, multiple lesions, and pulmonary embolism, while intrapulmonary factors include lung pneumonia, infection, aspiration and viruses [30]. In several papers, it has been reported that LPS intratracheal administration caused lung inflammation, principally to the alveolar epithelial cells, triggering lymphocytic and neutrophilic infiltration and panalveolitis [14]. Despite the fact that LPS animal models of ALI do not completely recapitulate the SARS-CoV2 pathology, LPS-induced ALI models may still be useful in studying cytokines storm release and the immune cells involvement as a complication of COVID-19. As a result, LPS has been widely used to induce ALI model [31]. Nevertheless, there is no effective therapy for ALI up to now; thus, elucidating the mechanism of pathogen infection-induced ALI will provide new ideas for clinical targeted drugs for the gene therapy treatment of ALI. Histopathological investigation by H & E staining, in association with MPO activity directly and W/D ratio indirectly, revealed that, in the current study, LPS-induced mouse ALI model was successfully obtained. In the present study, um-PEA oral administration, 1 h after LPS injection, significantly repaired the morphological and histological changes in lung tissue, as well as reduced lung MPO activity and W/D ratio after LPS-induced ALI in mice. Transepithelial neutrophil migration is an important feature of ALI, because neutrophils are the primary perpetrators of inflammation. Uncontrolled inflammatory responses are thought to play essential roles in the pathogenesis of ALI, which results in extensive neutrophil infiltration, massive release of inflammatory mediators, a widespread increase in capillary permeability and severe interstitial edema [32]. Moreover, um-PEA markedly alleviated the number of neutrophils in BALF, as well as the number of macrophages and immune cells. All these data are consistent with the MPO activity analysis, indicating that um-PEA administration markedly mitigated the neutrophils infiltration in injury induced by LPS challenge. Mast cells play an essential role in lung diseases; in fact, their activation leads to the consequent degranulation and release of inflammatory mediators [33]. Moreover it was seen that ALI, which can be a complication of intestinal ischemia reperfusion, occurred following pulmonary mast cell activation [34]. Among the various components released by the mast cell, there are histamine, cytokines, and several proteases, like chymase and tryptase. Histamine and tryptase are the main cytokines and chemokines released by mast cells, which subsequently induce inflammatory cells infiltration, promote cytokines generation, and increase vessel permeability, which, in turn, further facilitate mast cell migration and exacerbate inflammation [35,36]. An increase of chymase-positive mast cells number was seen in lungs with interstitial pneumonia [11]. In addition, an increase of chymase-positive mast cells number was reported in many studies with viral infections in the lungs or asthma, pulmonary hypertension, lung fibrosis and chronic obstructive pulmonary syndrome, similar to our study [37,38]. In the present study, treatment with um-PEA, after LPS-induced lung injury, was able to stabilize mast cell degranulation, and at the same time reduced chymase and tryptase activity after LPS-induced ALI. It is well known that many tissue responses to inflammatory injuries are activated and managed by cytokines. IL-6 is mainly produced by the innate immune system and is one of the first cytokines released in the acute phase of ARDS/ALI, and is followed by increases in the expression of IL-1β, IL-8 and TNF-α [39]. Amongst these, the most studied one is IL-6, which is increased both in mild and severe COVID-19 patients and correlated with the pulmonary infiltration area in patients with ARDS [40,41]. IL-18 is an important cytokine produced by macrophages in the early stages of viral infections and subsequently stimulates the production of IL-6 and IFN-γ, which are considered essential for optimal viral host defense [42,43]. The inhibitory action of PEA against the release of cytokines is widely documented in the literature. Several studies have shown that um-PEA, as well as many of its analogues such as Adelmidrol, N-Palmitoylethanolamine-oxazoline (PEA-OXA), the oxazoline of PEA or N-Palmitoyl-D-Glucosamine (PGA), were able to decrease the plasma levels of cytokines such as TNF-α and IL-1β [44,45,46,47,48]. Furthermore, um-PEA showed protective effect in lung; in fact, it was seen that um-PEA reduced inflammation in terms of cytokines release and immune cells infiltration in model of idiopathic pulmonary fibrosis induced by bleomycin intratracheal administration [21]. In the current study, the oral administration of um-PEA was able to reduce inflammatory cell infiltration in BALF, as well as decrease chemokines and cytokines expression induced by pulmonary injury. ELISA analysis of BALF also showed a reduced expression of the cytokines IL-6, TNF-α, IL-1β and IL-18, confirming the um-PEA action on inflammatory cytokines release. TLR4 is an important target recognized by LPS derived from Gram-negative bacteria [49]. The bind between LPS and TLR4 leads upstream to an activation of the transcription factor NF-κB, involved in the regulation of the production of pro-inflammatory mediators, such as IL-1β and IL-6, or TNF-α [50]. Although the mechanism of action of PEA is not yet well defined, it is well known that PEA was able to inhibit mast cell degranulation, through an Autacoid Local Injury Antagonism. At the basis of the several hypotheses advanced over the years on the mechanism of action of PEA, there is the capability to inhibit NF-kβ translocation into the nucleus, not directly but through a bind with receptor like PPARα [51]. As a result, treatment with um-PEA efficaciously blocked the phosphorylations of IκBα, NF-κB, which suggested that um-PEA suppressed excessive cytokine production by inhibiting NF-κB activation. With regard to the intracellular signaling involved in LPS-induced ALI, it is well known that increases in the production of inflammatory factors are associated with the phosphorylation of MAP kinases, such as p-38, ERK1/2 and JNK [52,53,54]. Moreover, previous study showed that PEA was able to reduce the p-ERK, as well as JNK/p38 levels after damage, in several models, like postoperative pain in vivo model or astrogliosis in vitro [55,56,57]. In our study, the p-p38 expression, such as p-ERK1/2, p-JNK and nuclear NF-κB, was significantly inhibited in ALI mice after the administration of um-PEA. In line with previous studies [57], these results suggested that um-PEA attenuates inflammation possibly through p38MAPK/NF-κB signaling pathway after LPS-induced pulmonary damage. Finally, it should be noted that, according to the 2012 Berlin definition, the term acute lung injury (ALI) as defined by the American–European Consensus Conference (AECC) has been removed, because it should instead talk of ALI non-ARDS or ARDS alone. Although the category of ALI non-ARDS is not explicitly described by the AECC, it has been used by many investigators [58]. Anyway, further studies are required to better understand the effect of PEA treatment in acute lung lesion. These results suggest that um-PEA can attenuate acute lung inflammation, reducing immune cell infiltration and cytokines release. Moreover, um-PEA exerted protective effect on LPS-induced ALI by suppressing the p38MAPK/NF-κB interation in a process that depends on the ERK1/2/JNK/p38/NFκB signaling axis.

## 4. Materials and Methods

### 4.1. Animals

Male CD1 mice (25–30 g, Envigo, Milan, Italy) were accommodated in a controlled location. They received food and water ad libitum, in vivo. The University of Messina Review Board for animal care (OPBA) approved the study (9 February 2017, 137/2017-pr). All in vivo experiments followed the new directives of the USA, Europe, Italy, and the ARRIVE guidelines.

### 4.2. Induction of Acute Lung Injury

For intratracheal instillation (i.t.), animals were anesthetized with isoflurane (2%), and LPS was instilled as previously described [59,60]. In particular, the trachea was exposed by a 1 cm long ventral midline cervical incision, and LPS or saline solution was instilled using a bent 27-gauge tuberculin needle. LPS and LPS+um-PEA groups received a single i.t. dose of Escherichia coli LPS (026: B6L3755, Sigma Aldrich, St. Louis, MO, USA) 5 mg.kg−1 suspended in saline solution (total volume = 0.05 mL per animal). All mice were sacrificed at 24 h post-induction, and BALF and lung tissues were collected for subsequent experiments. Ultramicronization of PEA was obtained as previously described by Impellizzeri et al. [61]. PEA was subjected to the air-jet milling technique, in which a coarse powder is slowly fed into a jet-mill apparatus endowed with a chamber of 300 mm in diameter that operates with “spiral technology” driven by compressed air. The high number of collisions that occur between particles as a result of the high level of kinetic—not mechanical—energy produces sub-micron-sized crystals [62].

### 4.3. Experimental Groups

Mice were randomized into the following experimental groups (*n* = 6):

- LPS + vehicle group. Mice received LPS i.t. and were treated with the vehicle (saline);
- LPS + um-PEA group. Mice received LPS i.t. and were treated with um-PEA 30 mg/kg (an animal of 25 g received 250 μL of um-PEA dissolved in carboxymethylcellulose CMC 1% and saline) 1 h after LPS i.t. instillation by oral gavage;
- Sham + vehicle group. Identical to the LPS + vehicle group, but animals received saline (0.9% *w*/*v*) i.t. instead of LPS;
- Sham + um-PEA group. Identical to the LPS + um-PEA group, but animals received saline (0.9% *w*/*v*) i.t. instead of LPS and were orally treated with um-PEA 30 mg/kg (an animal of 25 g received 250 μL of um-PEA dissolved in carboxymethylcellulose CMC 1% and saline) 1 h after LPS i.t. instillation by oral gavage.

Mice were euthanized by cervical dislocation 24 h after LPS instillation, and tissues were harvested for analyses of injury and inflammation. The dose of um-PEA was selected based on preliminary experiments performed in our laboratory.

### 4.4. Proteins Concentration and Cell Counts in BALF

Twenty-four hours after LPS instillation, mice were anesthetized with isoflurane and exsanguinated. The left lung was ligated, and the right lung was gently lavaged three times via the tracheal tube with a total volume of 0.7 mL of phosphate-buffered saline (PBS; Thermo Fisher Scientific, Waltham, MA, USA). Using Wright’s Giemsa stain (Nanjing Jiancheng, Nanjing, China), the leukocyte and macrophage populations present in BALF were counted. Briefly, the cell smear is stained with Wright-Giemsa solution for 1–2 min, after which it is rapidly dipped in PBS for 5 min, then rinsed with running distilled water for 30 min to decolor and finally air-dried. Differential cell counting was performed on each slide. BALF was performed 3 times with 1 mL of PBS in total and the BALF recovery ratio was approximately 80%, followed by centrifugation at 3000 rpm for 10 min at 4 °C. To determine the protein concentration and to measure the cytokines, the supernatants in BALF were analyzed by BCA Protein Assay Kit. The levels of IL-6 IL-1β, TNF-α and IL-18 in BALF were detected using ELISA kits (Dakewe, Shenzhen, China; Biosource International, Camarillo, CA, USA; BioLegend, San Diego, CA, USA).

### 4.5. Measurement of Lung Edema

The water content in the lungs was calculated as the wet weight/dry weight ratio of the tissue. The wet weight was obtained from tissue at sacrifice 24 h after instillation of LPS, while the dry weight was obtained by weighing the tissues after drying at 180 °C.

### 4.6. Histological Examination

After 24 h from LPS instillation lung tissue sample were collected and fixing in buffered formaldehyde solution (10% in PBS). For histological analyzes sections were stained with H & E, while for the determination of mast cell degranulation lung sections were stained with toluidine blue. All the histological images were taken using an Axiovision Zeiss (Milan, Italy) [63]. ALI scored was assessed as previously described [64] follows: alveolar congestion, hemorrhage, infiltration or aggregation of neutrophils in airspace or vessel wall, and thickness of alveolar wall/hyaline membrane formation. Each item score was scored on a five-point scale as follows: 0 = minimal damage, 1 = mild damage, 2 = moderate damage, 3 = severe damage, 4 = maximal damage.

### 4.7. MPO Assay

The determination of the MPO activity was carried out as described above, and was expressed in units per gram of wet tissue weight, defined as the quantity of enzyme capable of degrading 1 µmol of peroxide per minute at 37 °C [65,66].

### 4.8. Western Blot Analysis

Western blots were performed as described in our previous studies [67,68,69]. Briefly, lung tissues from each mouse were suspended in an extraction’s buffer containing 0.15 µM pepstatin A, 0.2 mM phenylmethylsulfonyl fluoride (PMSF), 1 mM sodium orthovanadate, and 20 µM leupeptin; homogenized at the highest setting for 2 min; and centrifuged at 1000× *g* for 10 min at 4 °C. Supernatants contain the cytosolic fractions, while the pellets represent the nuclear ones. Pellets were resuspended in a second buffer containing 150 mM sodium chloride (NaCl), 1% Triton X-100, 1 mM ethylene glycol tetraacetic acid (EGTA), 10 mM tris-chloridric acid (HCl) pH 7.4, 0.2 mM PMSF, 1 mM Ethylenediaminetetraacetic acid (EDTA), 0.2 mM sodium orthovanadate, and 20 µm leupeptin. After centrifugation at 4 °C and 15,000× *g* for 30 min, the nuclear proteins containing the supernatants were stored at −80 °C for further analysis. Specific primary antibody anti-IκB-α (SCB, 1:500 #sc1643), anti-NF-κB p65 (SCB; 1:500 #sc8008), anti-pJNK (1:500 Thermo Fisher), anti-pERK (SCB, 1:500 #sc7383), anti-p-p38 (SCB, 1:500 #sc7983) were mixed in 1× PBS, 5% *w*/*v* non-fat dried milk, and 0.1% Tween-20, and incubated at 4 °C overnight. Following this, blots were incubated with peroxidase-conjugated bovine anti-mouse IgG secondary antibody or peroxidase-conjugated goat anti-rabbit IgG (1:2000, Jackson Immuno Research, Cambridge, UK) for 1 h at room temperature. To verify that membranes were loaded with equal amounts of protein, they were also incubated with the antibody against Anti-β-actin or anti-lamin A/C (D.B.A., Milan, Italy). Signals were detected with enhanced chemiluminescence detection system reagent, according to the manufacturer’s instructions (Super-Signal West Pico Chemiluminescent Substrate, Pierce, (Waltham, MA, USA). The relative expression of the protein bands was quantified by densitometry with Bio-Rad ChemiDoc XRS software and standardized to β-actin levels. Images of blot signals (8-bit/600-dpi) were imported to analysis software (Image Quant TL, v2003, Marlborough, MA, USA).

### 4.9. Immunohistochemical Localization of Chymase and Tryptase

Immunohistochemical analysis was performed as previously described [70]. The sections were incubated overnight with primary antibodies anti-Chymase mouse polyclonal antibody (1:100 in PBS, *v*/*v*, Santa SCB, #sc59586), and anti-Tryptase mouse polyclonal antibody (1:100 in PBS, SCB #sc59587). All sections were washed with PBS and then treated as previously reported [68]. Five stained sections from each mouse were scored in a blinded fashion and observed using a Leica DM6 microscope (Leica Microsystems SpA, Milan, Italy) following a typical procedure [71]. The ImageJ IHC profiler plug-in was used for densitometric analysis. When this is selected, it automatically traces a histogram profile of the deconstructed 3, 3′-diaminobenzidine (DAB) and a corresponding score log is shown. The histogram profile is related to the positive pixel intensity value obtained [72].

### 4.10. Materials

All compounds used in this study were purchased from Sigma-Aldrich Company Ltd. (Milan, Italy) and were of the highest commercial grade available. Um-PEA was obtained from Epitech Group SpA.

### 4.11. Statistical Evaluation

All values in the figures and text are expressed as the mean ± standard error of the mean (SEM) of N number of animals. The results were analyzed by one-way ANOVA followed by a Bonferroni post hoc test for multiple comparisons.

## 5. Conclusions

Collectively, our data demonstrate that twenty-four hours after LPS i.t., um-PEA administration at dose of 30 mg/kg was able to attenuate the inflammatory response in terms of immune cells infiltration and cytokines release. Um-PEA reduced alterations in lung histopathology and decreased mast cell degranulation, as well as showed a decrease of IL-6, TNF-α, IL-1β and IL-18 expression. Therefore, we propose that um-PEA administered after LPS injection can be considered as a potential therapeutic approach in the treatment of acute pulmonary injury, and therefore may have clinical implications in conditions associated to lung inflammation with an altered cytokines profile such as COVID-19.

## Data Availability

Data available on request.
